# Modulation of cerebral activation strategies by training mode in stratified stroke cohorts: an fNIRS study

**DOI:** 10.3389/fnhum.2026.1767418

**Published:** 2026-03-18

**Authors:** Mengjian Qu, Huamin Li, Jia Fu, Changhao Le, Xiarong Huang, Xing Wen, Huali Tang, Fu Luo, Jun Zhou

**Affiliations:** 1 Department of Rehabilitation, Hengyang Medical School, The First Affiliated Hospital of University of South China, Hengyang, Hunan, China; 2 Rehabilitation Laboratory, Hengyang Medical School, The First Affiliated Hospital of University of South China, Hengyang, Hunan, China; 3 Department of Rehabilitation Sciences, The Hong Kong Polytechnic University, Kowloon, Hong Kong SAR, China

**Keywords:** functional near-infrared spectroscopy (fNIRS), mode sensitivity, precision rehabilitation, robot-assisted therapy, stroke rehabilitation

## Abstract

**Introduction:**

Robot-assisted training (RAT) exhibits inconsistent efficacy in post-stroke upper limb rehabilitation, with its underlying neural mechanisms remaining unclear. This study aimed to investigate how different therapy modes modulate cerebral activation strategies in distinct subgroups of stroke patients.

**Methods:**

We utilized functional near-infrared spectroscopy (fNIRS) to investigate differences in cortical activation strategies, specifically the response sensitivity to various robotic training modes, by stratifying forty-one patients based on functional level, disease chronicity, and hemiplegic side. In a single session, each participant underwent four RAT modes (Passive, Assistive-active, Active, and Mirror) while a 48-channel fNIRS system monitored cortical activation.

**Results:**

Our results revealed no statistically significant differences in global mean activation intensity between any of the subgroups (*p* > 0.05). Instead, the core finding was a clear dichotomy in neural strategy: low-function, subacute, and left-hemiplegia groups were highly “mode-sensitive,” exhibiting significant changes in brain activation across different training modes (e.g., Active vs. Mirror, *p* < 0.05). Conversely, high-function, chronic, and right-hemiplegia groups were “mode-consolidated,” demonstrating a stable activation pattern with almost no significant differences among the active modes.

**Discussion:**

We conclude that the core neural mechanism of post-stroke recovery is characterized not by simple changes in activation intensity, but by a strategic evolution from a flexible, cue-dependent “mode-sensitive” state to a more automated “mode-consolidated” state. This distinction provides a robust neurophysiological rationale for personalizing rehabilitation, enabling clinicians to strategically match therapeutic stimuli to a patient's specific neural profile—applying diversified training to “mode-sensitive” patients and high-load challenges to “mode-consolidated” patients—to break through rehabilitation plateaus.

## 1 Introduction

Stroke remains a leading cause of long-term disability worldwide, with approximately 80% of survivors experiencing some degree of functional impairment (Feigin et al., 2025; Niu et al., 2025). Beyond the physical toll, the prolonged dependency of survivors exerts a profound impact on modern healthcare systems, necessitating resource-intensive long-term care. Pathophysiologically, the cerebral injury triggers a complex cascade of events, including neuroinflammation and oxidative stress, which collectively drive neuronal cell death and functional deficits (Purrahman et al., 2023). While these molecular mechanisms underpin the initial tissue damage, the restoration of motor function depends heavily on the brain's intrinsic capacity for network reorganization and plasticity (Stinear et al., 2020). Among these sequelae, upper limb hemiparesis is particularly pervasive and debilitating, profoundly compromising an individual's capacity to perform essential activities of daily living (ADL) and consequently diminishing their independence and quality of life. The prolonged and often incomplete recovery of motor function not only imposes a significant psychological and physiological burden on patients but also creates substantial economic and caregiving challenges for their families and society (Langhorne et al., 2011). Therefore, optimizing therapeutic strategies to enhance upper limb motor recovery is a paramount objective in the field of neurorehabilitation.

The foundation of motor rehabilitation is built upon a spectrum of training modes, each with a distinct theoretical basis and clinical application. These range from Passive movement, which relies on an external force to maintain joint mobility and provide sensory input, to Active movement, which requires the patient's own volitional effort to drive motor commands and engage cortical networks. Intermediary modes like Assistive-Active training bridge this gap, pairing a patient's intent with external support, while other approaches like Mirror Therapy utilize visual illusion to modulate cortical activity. The principle of neuroplasticity—the brain's intrinsic capacity for reorganization—is the common target of all these modes (Wu et al., 2021). It is now well-established that high-dosage, high-repetition, and task-oriented training are essential to effectively drive this plasticity and promote functional recovery (Kwakkel et al., 2008).

However, rigorously comparing the neurophysiological effects of these different modes in a clinical setting presents a significant methodological challenge. Conventional manual therapy, while crucial, is inherently variable and subject to therapist fatigue, making it difficult to deliver the standardized, high-intensity dosage required for robust scientific inquiry. It is in this context that robotic platforms serve as indispensable scientific tools (Park et al., 2020). By providing precisely controlled, repeatable, and quantifiable delivery of various training modes—from purely passive motion to active resisted tasks—robotic systems allow us to isolate the variable of interest: the training mode itself (Aguirre-Ollinger et al., 2024). This enables a systematic investigation into how the brain responds to these distinct sensorimotor inputs, an endeavor that would be unfeasible with conventional methods alone.

Despite the standardization offered by such platforms, clinical outcomes remain highly variable (Rodgers et al., 2019; Xie et al., 2022). This inconsistency strongly suggests that the central issue is not the delivery system, but rather a “one-size-fits-all” application of these training modes to a profoundly heterogeneous stroke population. Patients differ vastly in their functional status, stage of recovery (chronicity), and the neuroanatomical characteristics of their lesion (Mehrholz et al., 2020). It is highly probable that a training mode that is beneficial for a patient in the subacute phase may be ineffective or even counterproductive for a patient in the chronic phase. This highlights an urgent need to move beyond monolithic treatment paradigms and toward a precision rehabilitation framework, where therapeutic strategies are tailored to specific, neurobiologically-defined patient subgroups (Xie et al., 2022).

To build such a framework, we must first understand the distinct neural processes that different training modes induce in different types of patients. Functional near-infrared spectroscopy (fNIRS) is an ideal neuroimaging modality for this purpose. As a non-invasive, portable, and motion-tolerant technology (Scholkmann et al., 2014), fNIRS allows for the real-time monitoring of cortical hemodynamics during the performance of functional tasks in ecologically valid settings (Rahman et al., 2020). This provides a direct window into the brain's strategic response to therapy as it unfolds, making it perfectly suited for integration with robotic rehabilitation paradigms.

This study proposes that the critical difference among patient subgroups lies not in the overall magnitude of brain activation, but in the nature of their underlying activation strategy. We introduce the concept of “mode sensitivity” to define this strategic difference, characterizing it as the degree to which an individual's cortical activation patterns are modulated by changes in the therapeutic training mode. This conceptual framework is grounded in established principles of post-stroke neural recovery and hemispheric specialization, leading to two primary hypotheses.

First, based on the dynamic evolution of neural recovery—from a diffuse, bilateral state to a more focal, efficient network (Feydy et al., 2002; Takeda et al., 2007)—we hypothesize that patients in phases of higher neuroplasticity (i.e., the subacute stage and those with lower functional levels) will exhibit greater “mode sensitivity.” Their brains are in an active state of exploration, highly dependent on external cues to guide network reorganization. Second, drawing from the principles of hemispheric specialization (Zemke et al., 2003; Riecker et al., 2010), we hypothesize that patients with right hemisphere lesions (left hemiplegia) will demonstrate higher “mode sensitivity,” reflecting a more extensive, strategic exploratory process orchestrated by the intact, dominant left hemisphere.

Therefore, the primary objective of this study is to utilize a robotic platform as a tool for standardized delivery to systematically investigate how cortical activation strategies are modulated by distinct training modes (Passive, Assistive-Active, Active, and Mirror) in subgroups of stroke patients stratified by hemiplegic side, functional level, and disease chronicity. By testing the concept of “mode sensitivity,” we aim to uncover fundamental neural signatures of the recovery process, thereby laying the neuroscientific groundwork for a more precise and effective paradigm of personalized rehabilitation.

## 2 Materials and methods

### 2.1 Participants

A total of 41 individuals with post-stroke upper limb motor dysfunction were recruited for this study. Inclusion criteria were: (1) a first-ever unilateral stroke confirmed by computed tomography (CT) or magnetic resonance imaging (MRI); (2) an upper limb functional level rated between 3 and 6 on the Functional Test for the Hemiplegic Upper Extremity, Hong Kong version (FTHUE-HK); (3) a Mini-Mental State Examination (MMSE) score of ≥20, ensuring the ability to understand and follow experimental instructions; and (4) right-handedness as determined prior to the stroke event. Exclusion criteria included a history of other neurological or psychiatric disorders, severe cognitive or aphasic impairments, and any contraindications for fNIRS measurement, such as cranial defects or significant head skin lesions.

**Figure 1:**
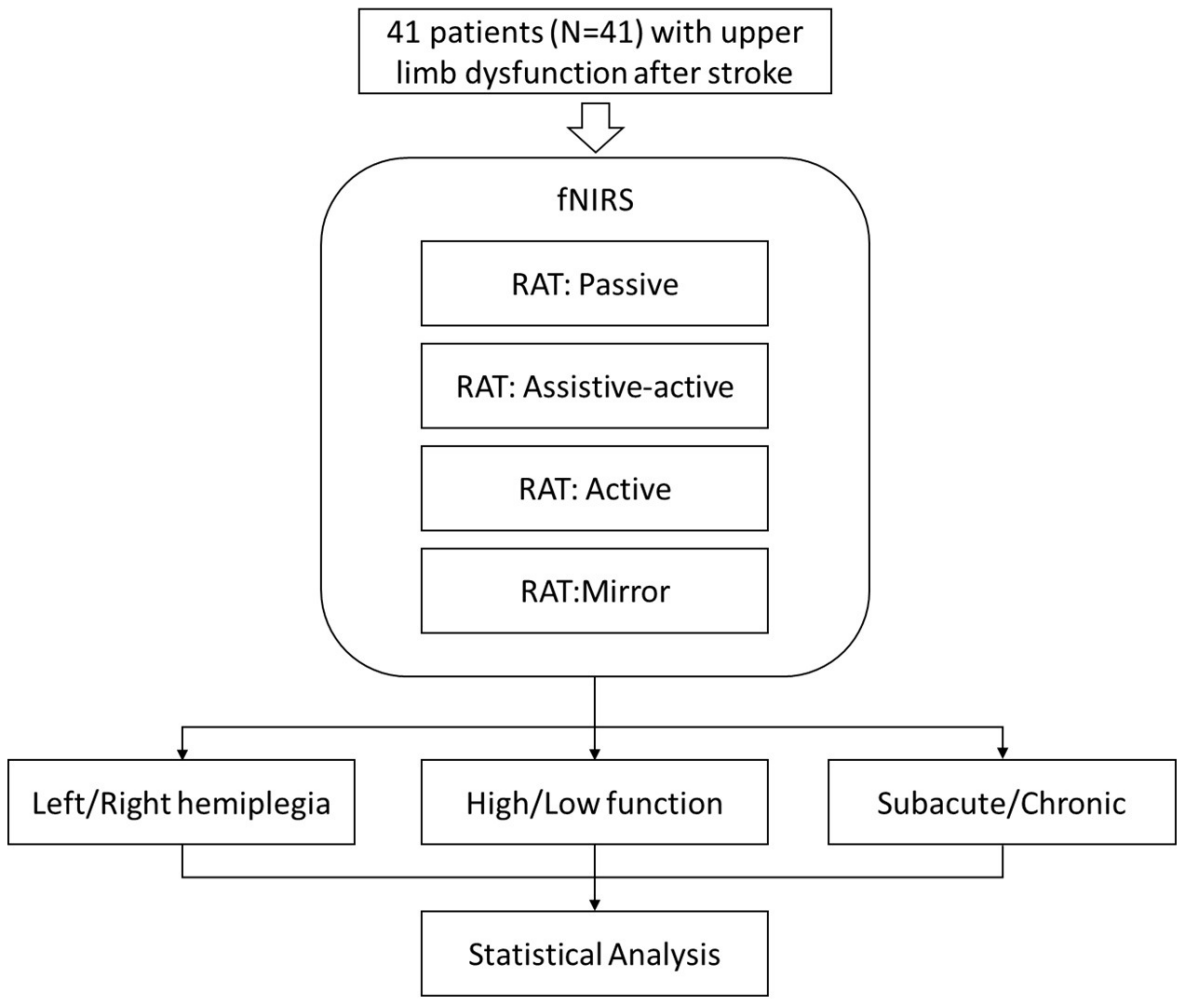
Flowchart of the experiment.

Participants were stratified into three pairs of subgroups for analysis: (1) hemiplegic side: left hemiplegia (*n* = 21) vs. right hemiplegia (*n* = 20); (2) disease chronicity: subacute phase (≤6 months post-stroke, *n* = 28) vs. chronic phase (>6 months post-stroke, *n* = 13); and (3) functional level: high-function (FTHUE-HK score 5–6, *n* = 12) vs. low-function (FTHUE-HK score 3–4, *n* = 29). The study protocol was approved by the Ethics Committee of the First Affiliated Hospital of Nanhua University (Approval No. 2024KS-KF-28-02) and was registered with the Chinese Clinical Trial Registry (ChiCTR2500096992). All participants provided written informed consent prior to their inclusion in the study. Flowchart of the experiment was elaborated in Figure 1.

### 2.2 Rehabilitation robot and training modes

The study utilized the Wisebot-X5, a three-dimensional (3D) upper limb exoskeleton rehabilitation robot (Shenzhen Huaquejing Medical Technology Co., Ltd., China). This device is designed to enable multi-joint arm movements within a gamified 3D workspace, simulating functional tasks. The robot was configured to deliver four distinct training modes, each with precise operational parameters. The general range of motion for the tasks included shoulder horizontal abduction up to 90° and adduction to 40°, with shoulder forward flexion from 45 to 130°.

The four training modes were defined as follows: (1) passive mode: the robot fully drove the participant's affected limb through a predefined trajectory at a constant angular velocity of 15°/s, requiring the participant to remain relaxed. (2) Assistive-active mode: the participant initiated the movement, and the robot provided an assistive torque of 2 N·m only when the participant's voluntary effort was insufficient to complete the task. (3) Active mode: the participant volitionally drove the robotic arm to complete the task against a constant resistance of 1 kg. (4) Mirror mode: a body-sensing camera captured the real-time motion of the participant's unaffected limb, and the robot drove the affected limb to perform a symmetrical, mirrored movement at a maximum velocity of 60°/s.

### 2.3 Experimental design and procedure

**Figure 2:**
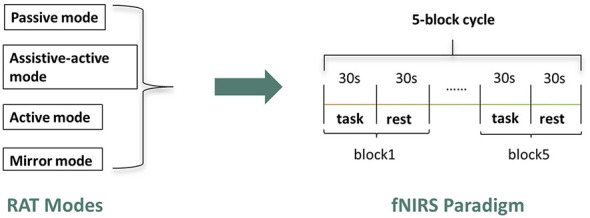
Experimental design.

This study employed a cross-sectional, repeated-measures design in which each participant underwent all four robotic training modes within a single experimental session (Figure 2). The presentation order of the four modes was pseudo-randomized across participants to mitigate potential order effects. For each training mode, a classic block design paradigm was implemented. This paradigm consisted of five repeated cycles, with each cycle comprising a 30-s task execution period followed by a 30-s resting period. Prior to the first task block, a 30-s baseline recording was acquired to serve as a reference for subsequent activation analysis.

To ensure anatomical accuracy for group-level analysis, the 3D coordinates of each source and detector were recorded for every participant using a 3D digitizer. These coordinates were subsequently transformed into the Montreal Neurological Institute (MNI) standard coordinate space, allowing for precise anatomical localization of the measured cortical activity. Signal quality was visually inspected prior to each recording to ensure good optode-scalp contact.

### 2.4 fNIRS data acquisition

**Figure 3:**
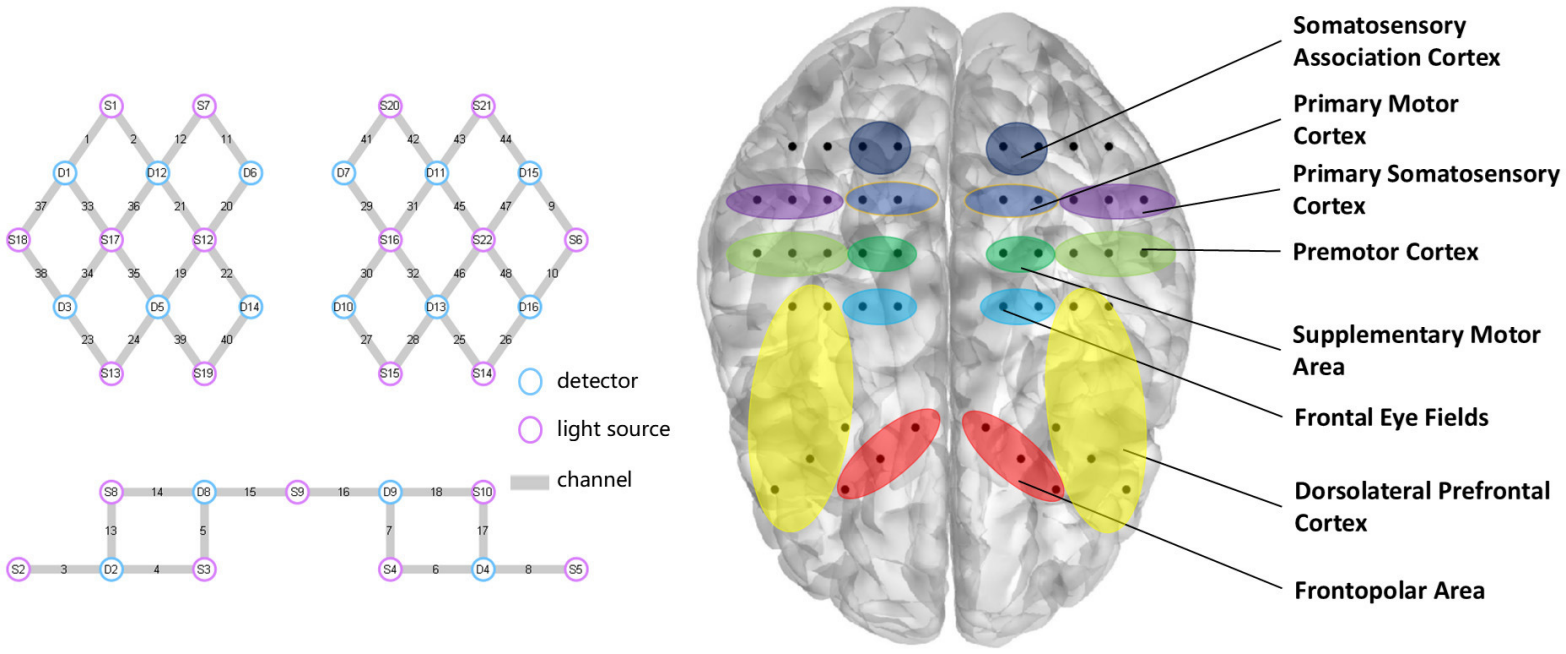
Arrangement of the channels and distribution of ROI.

Cortical hemodynamic responses were continuously monitored using a 48-channel continuous-wave fNIRS system (NirSmart, DanYang HuiChuang Medical Equipment Co., Ltd., China). The system was configured with 21 light sources and 16 detectors, utilizing two wavelengths of near-infrared light (730 and 850 nm) at a sampling rate of 10 Hz. The optodes were arranged with a standard source-detector separation of 30 mm and were securely mounted in a specialized cap positioned according to the international 10–20 system. The cap placement ensured comprehensive coverage of bilateral motor-related cortices (Table 1), including the primary motor cortex (M1), premotor cortex (PMC), supplementary motor area (SMA), primary somatosensory cortex (PSC), dorsolateral prefrontal cortex (DLPFC), frontopolar area (FP), frontal eye fields (FEF), and somatosensory association cortex (SAC). The arrangement of the channels and the detailed annotation of the brain's regions of interest (ROI) based on Brodmann area were elaborated in Figure 3.

**Table 1:** Detailed annotation of ROI based on Brodmann area and corresponding functions.

**ROI**	**Channel**	**Function**
Left M1	CH29, CH31	Motor execution: generates the final motor commands for direct control of fine movements of the contralateral side of the body
Right M1	CH20, CH21	
Left SMA	CH30, CH32	Motor planning and sequencing: internally plans, prepares, and sequences complex movements, especially crucial for bimanual coordination
Right SMA	CH19, CH22	
Left PMC	CH10, CH46, CH48	Movement preparation and guidance: prepares for movements guided by external (e.g., visual, auditory) cues and transforms sensory information into motor commands
Right PMC	CH34, CH35, CH38	
Left PSC	CH33, CH36, CH37	Primary sensory processing: receives and processes basic sensory information (touch, pressure, temperature, proprioception) from the contralateral (right) side of the body
Right PSC	CH9, CH45, CH47	
Left FP	CH6, CH7, CH16	High-Level Cognitive Planning: Responsible for the highest level of goal setting, long-term planning, decision-making, and exploratory behavior, indirectly influencing motor strategy selection
Right FP	CH4, CH5, CH15	
Left DLPFC	CH8, CH17, CH18, CH25, CH26	Working memory and executive function: maintains and manipulates relevant information (e.g., task rules) during movement, monitors performance, and inhibits inappropriate actions
Right DLPFC	CH3, CH13, CH14, CH23, CH24	
Left FEF	CH27, CH28	Eye-hand coordination and attentional guidance: controls voluntary eye movements to locate visual targets and directs spatial attention toward the goal of the movement
Right FEF	CH39, CH40	
Left SAC	CH11, CH12	Higher-order sensory integration: integrates various sensory inputs from the PSC to form a detailed perception of objects and one's own limb position
Right SAC	CH41, CH42	

### 2.5 fNIRS data analysis

All fNIRS data were processed and analyzed using the NirSpark software (v1.8.9) (DanYang HuiChuang Medical Equipment Co., Ltd., Danyang, China). (Chu et al., 2023). The preprocessing pipeline began with the identification and exclusion of channels with poor signal quality, defined by a coefficient of variation greater than 20%. The raw light intensity data from the remaining channels were then converted into changes in optical density. Subsequently, a band-pass filter with a cutoff range of 0.01–0.2 Hz was applied to remove physiological noise, such as cardiac pulsations and respiratory signals, as well as low-frequency signal drift. Motion artifacts were detected and corrected using a spline interpolation algorithm.

Finally, the preprocessed optical density data were converted into concentration changes of oxyhemoglobin (ΔHbO) and deoxyhemoglobin using the modified Beer-Lambert Law (MBLL). For all subsequent statistical analyses, we focused on ΔHbO signals, as they are considered to have a higher signal-to-noise ratio and a stronger correlation with task-related neural activity.

### 2.6 Statistical analysis

All statistical analyses were conducted using Python, with the significance level set at α = 0.05. The Shapiro-Wilk test was first applied to all continuous variables to assess for normality, guiding the selection of either parametric or non-parametric tests. To determine task-related cortical activation, the mean ΔHbO values during task blocks were compared against the baseline using one-sample t-tests (for normally distributed data) or Wilcoxon signed-rank tests (for non-normally distributed data) for each ROI. The resulting *p*-values were corrected for multiple comparisons using the Benjamini–Hochberg false discovery rate (FDR) procedure.

To compare overall activation levels between the stratified subgroups (e.g., high- vs. low-function), independent samples *t*-tests or Mann–Whitney *U* tests were employed. Analysis of covariance (ANCOVA) was used to control for the effects of confounding baseline variables, such as age, where significant differences between groups were identified. To assess “mode sensitivity” within each subgroup, a repeated measures ANOVA or a non-parametric Friedman test was conducted to identify significant differences in activation across the four training modes. Significant main effects were followed by *post-hoc* paired t-tests or Wilcoxon signed-rank tests with FDR correction. To explore the relationship between neural strategies and clinical outcomes, Spearman's rank correlation analysis was used to test the association between a quantified “Mode Sensitivity Index” (defined as the global mean activation difference between the Active and Passive modes) and both the FTHUE-HK score and the time since stroke.

## 3 Results

### 3.1 Participant characteristics and baseline demographics

The demographic and clinical characteristics of the 41 participants are presented in Table 2. As per the stratification criteria, the chronic group had a significantly longer time since stroke compared to the subacute group (*p* < 0.001). Furthermore, the high-function group was significantly older than the low-function group (*p* = 0.016), and the age difference between the subacute and chronic groups approached statistical significance (*p* = 0.056). An analysis of covariance (ANCOVA), controlling for age, confirmed that there were no significant main effects of functional level (*F*(1, 38) = 1.563, *p* = 0.219) or disease chronicity (*F*(1, 38) = 1.743, *p* = 0.195) on global mean activation during the active training mode. This finding indicates that the observed differences between subgroups were not attributable to overall neural effort but rather to the underlying activation strategies.

**Table 2:** Baseline demographic and clinical characteristics of participants.

**Characteristic**	**All patients (*N* = 41)**	**Left hemiplegia (*n* = 21)**	**Right hemiplegia (*n* = 20)**	***p*-value^a^**	**High-function (*n* = 12)**	**Low-function (*n* = 29)**	***p*-value^b^**	**Subacute phase (*n* = 28)**	**Chronic phase (*n* = 13)**	***p*-value^c^**
Age (years), Mean ± SD	53.8 ± 12.1	53.4 ± 10.5	54.1 ± 13.8	0.863	60.2 ± 9.6	51.1 ± 12.2	0.016	51.6 ± 13.0	58.4 ± 8.6	0.056
Time since stroke (days), Mean ± SD	162.4 ± 164.1	168.4 ± 169.8	156.4 ± 162.4	0.409	172.6 ± 203.4	158.1 ± 148.3	0.281	83.5 ± 35.8	346.6 ± 198.8	0.000
FTHUE-HK, Mean ± SD	4.0 ± 1.1	3.8 ± 1.0	4.3 ± 1.2	0.241	5.5 ± 0.5	3.4 ± 0.5	0.000	4.0 ± 1.1	4.0 ± 1.2	0.879
FMA-UE, Mean ± SD	26.6 ± 8.9	24.4 ± 7.2	28.8 ± 10.1	0.139	35.9 ± 7.6	23.1 ± 6.6	0.000	28.0 ± 9.2	23.2 ± 7.5	0.108
**Gender**, ***n*** **(%)**										
Male	26 (63.4)	13 (61.9)	13 (65.0)	1.000	9 (75.0)	17 (58.6)	0.480	16 (57.1)	10 (76.9)	0.305
Female	15 (36.6)	8 (38.1)	7 (35.0)		3 (25.0)	12 (41.4)		12 (42.9)	3 (23.1)	
**Stroke type**, ***n*** **(%)**										
Hemorrhagic	19 (48.7)	9 (45.0)	10 (52.6)	0.752	6 (54.5)	13 (46.4)	0.731	15 (57.7)	4 (30.8)	0.176
Ischemic	20 (51.3)	11 (55.0)	9 (47.4)		5 (45.5)	15 (53.6)		11 (42.3)	9 (69.2)	
Not specified	0 (0.0)	0 (0.0)	0 (0.0)		0 (0.0)	0 (0.0)		0 (0.0)	0 (0.0)	
**Phase**, ***n*** **(%)**										
Subacute	28 (68.3)	14 (66.7)	14 (70.0)	1.000	8 (66.7)	20 (69.0)	1.000	28 (100.0)	0 (0.0)	–
Chronic	13 (31.7)	7 (33.3)	6 (30.0)		4 (33.3)	9 (31.0)		0 (0.0)	13 (100.0)	
**Function level**, ***n*** **(%)**										
High-function	12 (29.3)	4 (19.0)	8 (40.0)	0.181	12 (100.0)	0 (0.0)	–	8 (28.6)	4 (30.8)	1.000
Low-function	29 (70.7)	17 (81.0)	12 (60.0)		0 (0.0)	29 (100.0)		20 (71.4)	9 (69.2)	

^a^*p*-value for comparison between Left hemiplegia (*n* = 21) and Right hemiplegia (*n* = 20).

^b^*p*-value for comparison between High-function (*n* = 12) and Low-function (*n* = 29).

^c^*p*-value for comparison between Subacute phase (*n* = 28) and Chronic phase (*n* = 13).

### 3.2 Influence of hemiplegic side on brain activation strategies

**Figure 4:**
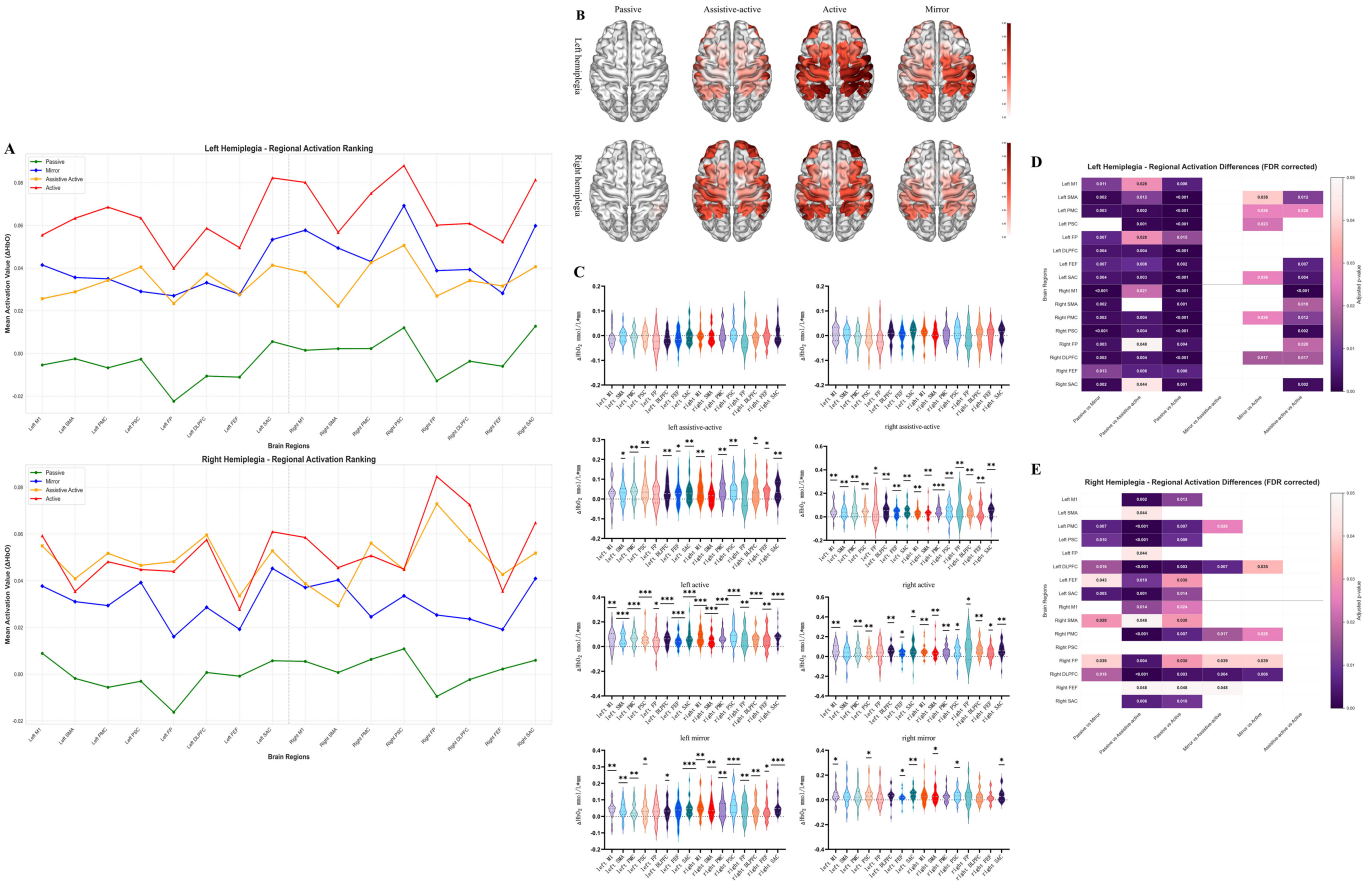
**(A)** Average activation values of brain regions for each training mode in the left/right hemiplegia group; **(B)** brain activation topographic maps for each RAT training mode in left/right hemiplegic patients; **(C)** activation status of brain regions for each RAT training mode in left/right hemiplegic patients; **(D)** comparison of brain region activation differences among each mode in left hemiplegia patients; **(E)** comparison of brain region activation differences among each mode in right hemiplegia patients.*p < 0.05; **p < 0.01; ***p < 0.001.

The two hemiplegia groups exhibited distinct activation characteristics during the different training modes (Figures 4B, C). For the left hemiplegia group (right hemisphere lesion), the passive mode did not elicit significant activation in any of the measured ROIs (*p* > 0.05). In contrast, both the assistive-active and active modes induced robust and widespread significant activation across all ROIs, including M1, PMC, and SMA (*p* < 0.05). The mirror mode also resulted in significant activation in most ROIs, with the exception of the left FP and FEF. For the right hemiplegia group (left hemisphere lesion), the passive mode was similarly non-activating (*p* > 0.05). The assistive-active and active modes significantly activated all ROIs (*p* < 0.05), though the active mode showed a slightly more restricted pattern, failing to activate the left FP and SMA. The mirror mode in this group induced a much more localized activation pattern, significant only in the left M1, left FEF, right SMA, and bilateral PSC and SAC (*p* < 0.05).

To assess for differences in overall neural effort, we compared the global mean activation between the left and right hemiplegia groups specifically during the active mode. An independent samples t-test revealed no statistically significant difference in activation magnitude between the two groups (*p* > 0.05, Supplementary Table 2a) (Figure 4A). Furthermore, we tested the classic hypothesis of a contralesional activation bias. Our intra-hemispheric analysis in both the left and right hemiplegia groups found no significant asymmetry in activation between the unaffected and affected hemispheres, either at the whole-hemisphere level or within corresponding ROIs (*p* > 0.05, Supplementary Tables 2b, c). This null finding suggests that a simple contralesional dominance model does not adequately describe the activation patterns in this heterogeneous sample.

The primary distinction between the groups was revealed in their response to changing training modes. The left hemiplegia group demonstrated a highly “mode-sensitive” profile. As shown in the heatmap in Figure 4D, there were significant and widespread activation differences between the passive mode and all three active-participation modes across nearly all ROIs. Crucially, significant differences were also observed among the active modes (e.g., Active vs. Assistive-Active; Mirror vs. Active), indicating a dynamic neural strategy that was finely tuned to the specific demands of each mode. Conversely, the right hemiplegia group exhibited a “mode-consolidated” strategy. Although their active modes were significantly different from the passive mode, the number of brain regions showing these differences was considerably smaller (Figure 4E). More importantly, there were almost no significant activation differences among the three active-participation modes. This suggests a more fixed, less adaptable neural activation pattern.

### 3.3 Influence of functional level on activation strategies

**Figure 5:**
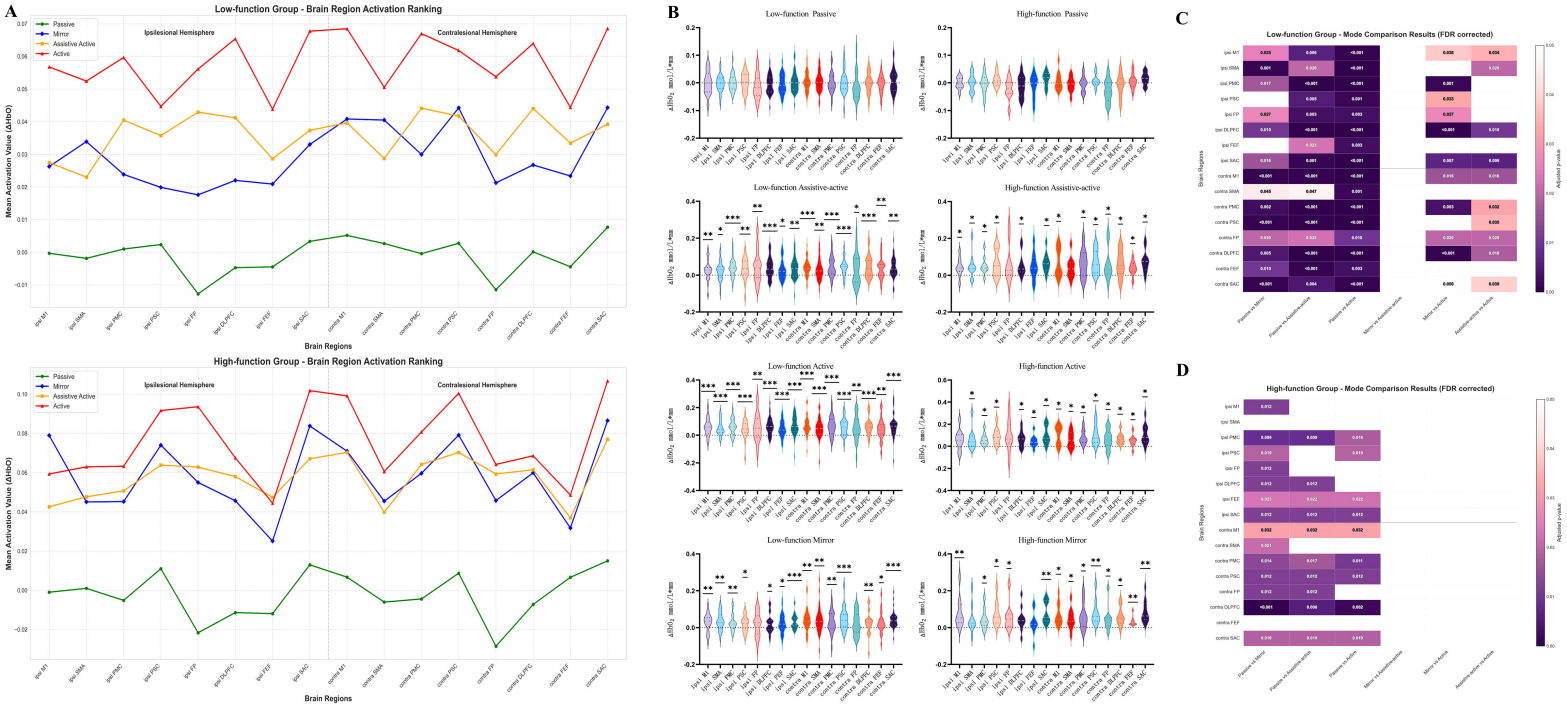
**(A)** Average activation values of brain regions for each training mode in the low/high function groups; **(B)** activation of brain regions in each RAT training mode for patients in the low/high function groups; **(C)** comparison of brain region activation differences among each mode in the low-function group; **(D)** comparison of brain region activation differences among each mode in the high-function group. *p < 0.05; **p < 0.01; ***p < 0.001.

Activation patterns also varied markedly with functional level (Figure 5B). In the low-function group, the passive mode was non-activating (*p* > 0.05). In contrast, the assistive-active, active, and mirror modes all induced widespread, significant activation across nearly all ROIs (*p* < 0.05). In the high-function group, the passive mode was also non-activating. However, the active-participation modes recruited a more focused neural network; specifically, the assistive-active mode failed to activate the ipsilesional FP, FEF, and contralateral SMA, the active mode failed to activate the ipsilesional FP and M1, and the mirror mode failed to activate the ipsilesional FEF, DLPFC, and SMA (all *p* > 0.05).

Consistent with the findings from the hemiplegic side analysis, there was no statistically significant difference in global mean activation during the active mode between the high- and low-function groups (*p* > 0.05, Supplementary Table 3a) (Figure 5A). Similarly, intra-hemispheric analyses within both the high- and low-function groups revealed no significant activation asymmetry between the ipsilesional and contralesional hemispheres (*p* > 0.05, Supplementary Tables 3b, c). These null findings further support that the key distinction between these groups is strategic rather than quantitative.

The core difference was again found in mode sensitivity. The low-function group was highly mode-sensitive, showing significant activation differences between the passive mode and all active modes across a vast array of ROIs (Figure 5C). Significant distinctions were also present among the active modes, particularly between the active mode and the other two (mirror and assistive-active), evident in key motor ROIs such as bilateral M1, DLPFC, and SAC. In striking contrast, the high-function group was “mode-insensitive.” As depicted in Figure 5D, there were no statistically significant differences in cortical activation among any of the three active-participation modes, indicating a stable and automated neural strategy.

### 3.4 Influence of disease chronicity on activation strategies

**Figure 6:**
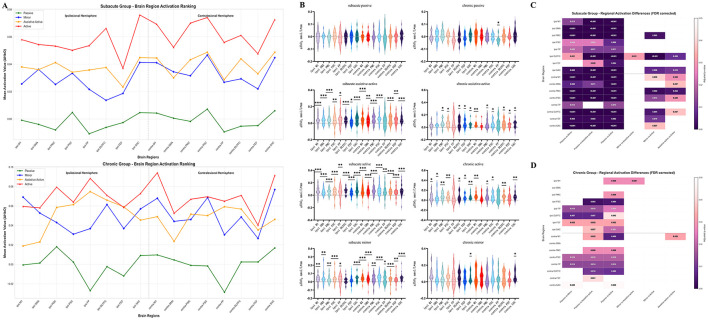
**(A)** Average activation values of brain regions in each training mode for the subacute/chronic phase group; **(B)** activation of brain regions in each RAT training mode for patients in the subacute/chronic phase group; **(C)** comparison of brain region activation differences among each training mode in the subacute group; **(D)** comparison of brain region activation differences among each training mode in the chronic phase group. *p < 0.05; **p < 0.01; ***p < 0.001.

Stratification by disease chronicity revealed a similar trend (Figure 6B). In the subacute group, the passive mode was non-activating, whereas all three active-participation modes elicited robust and significant activation across almost all measured ROIs (*p* < 0.05). In the chronic group, a more efficient activation pattern was observed. While the passive mode remained non-activating, the active modes required the engagement of a smaller set of brain regions. Specifically, during the assistive-active and active modes, the ipsilesional M1, contralateral FP, and SMA were not significantly activated, and during the mirror mode, only bilateral SAC showed significant activation (all *p* > 0.05).

Replicating the pattern from previous analyses, there was no significant difference in global mean activation during the active mode between the subacute and chronic groups (*p* > 0.05, Supplementary Table 4a) (Figure 6A). Intra-hemispheric analyses also showed no significant activation lateralization in either the subacute or chronic groups (*p* > 0.05, Supplementary Tables 4b, c) (Figure 6A).

The analysis of between-mode differences demonstrated a clear evolution of neural strategy over time. Subacute patients were highly mode-sensitive, with their cortical activation patterns being significantly modulated by the specific training mode (Figure 6C). They showed widespread, significant activation differences between passive and active modes, and also among the different active modes (e.g., in bilateral DLPFC, contralateral M1, and PMC between Active vs. Mirror modes). This dynamic response diminished in the chronic phase. Chronic patients displayed a marked reduction in mode sensitivity, with fewer brain regions showing differences between passive and active states and substantially fewer significant differences among the active modes themselves (Figure 6D), indicating a progression toward a more consolidated and less plastic activation strategy.

### 3.5 Relationship between mode sensitivity and clinical variables

**Figure 7:**
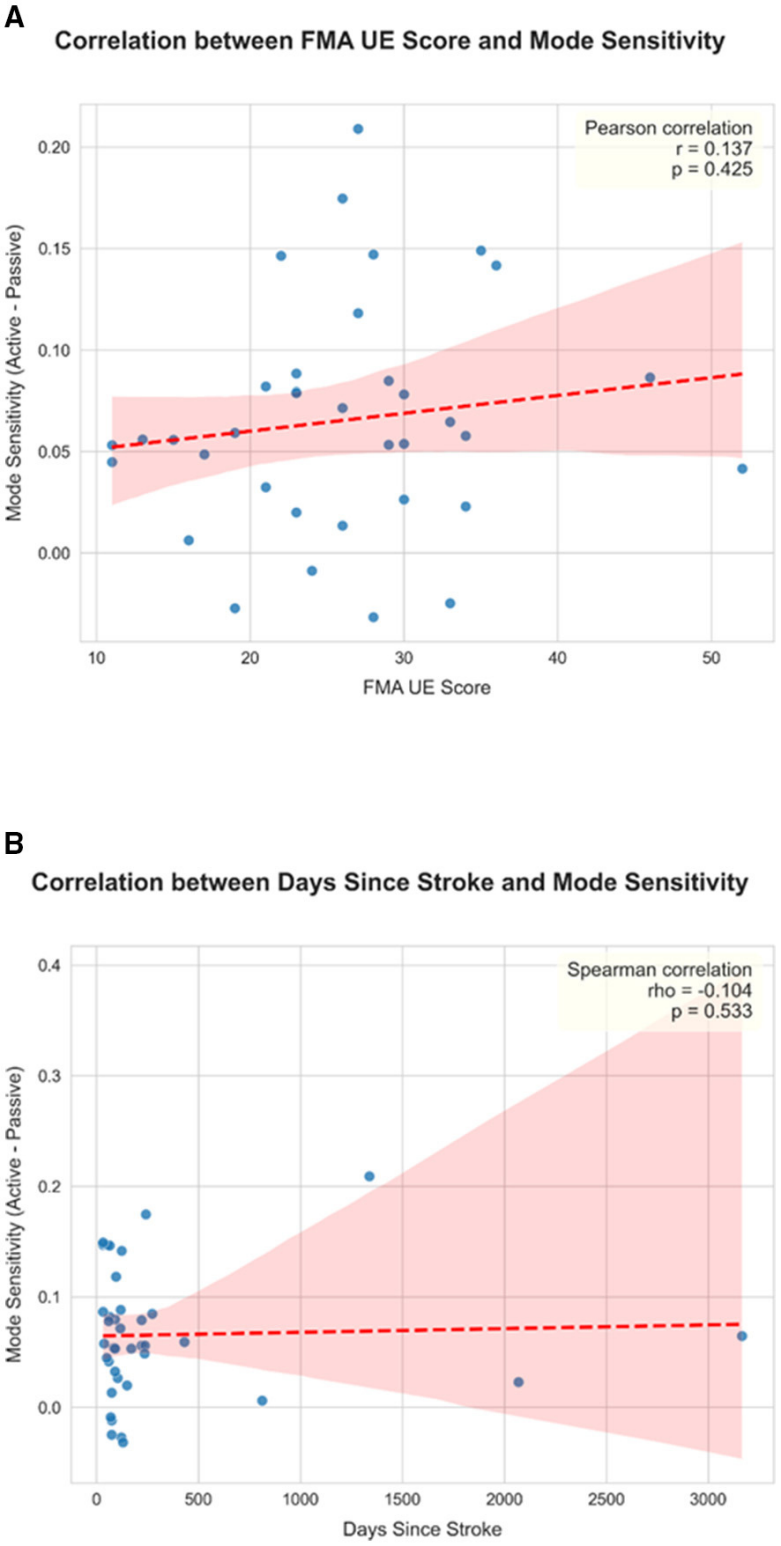
Correlation between FMA-UE/Days since stroke and Mode sensitivity. **(A)** Scatter plot illustrating the relationship between mode sensitivity and upper limb functional level. Upper limb function was assessed using the FMA-UE Score. Mode sensitivity was quantified as the difference in global mean activation between the Active and Passive modes (ΔHbO). Each point represents an individual participant (*n* = 40). The red dashed line indicates the line of best fit from the regression analysis, and the shaded area represents the 95% confidence interval. A weak positive trend was observed, but Spearman's rank correlation analysis did not find a statistically significant relationship between the two variables (rho = 0.137, *p* = 0.425). **(B)** Scatter plot illustrating the relationship between mode sensitivity and time since stroke. Mode sensitivity was quantified as the difference in global mean activation between the Active and Passive modes (ΔHbO). Each point represents an individual participant (*n* = 40). The red dashed line indicates the line of best fit from the regression analysis, and the shaded area represents the 95% confidence interval. Spearman's rank correlation analysis revealed no significant relationship between the two variables (rho = −0.104, *p* = 0.533).

To investigate if the “mode sensitivity” strategy existed on a continuum that correlated with clinical metrics, an exploratory analysis was performed. A “Mode Sensitivity Index” was calculated as the difference in global mean ΔHbO between the Active and Passive modes. Spearman's rank correlation analysis revealed a weak, non-significant positive trend between this index and the FMA-UE functional score (rho = 0.137, *p* = 0.425) and no significant relationship with the time since stroke (rho = −0.104, *p* = 0.533; Figure 7). The absence of a strong linear correlation suggests that “mode sensitivity” may represent a distinct categorical state of neural organization rather than a continuous variable that scales directly with function or recovery time.

## 4 Discussion

### 4.1 Principal finding: a paradigm shift from activation magnitude to activation strategy

A key finding of this study was the consistent lack of significant differences in the overall magnitude of global cortical activation between groups stratified by hemiplegic side, functional level, or disease chronicity. This robust null result serves as the central pivot for our research, deliberately shifting the scientific focus from merely quantifying the magnitude of activation to qualitatively assessing the activation strategy. It provides strong evidence for our core thesis: the critical distinction in post-stroke neural recovery lies not in the quantitative “volume” of neural resources recruited, but in the qualitative “strategy” by which those resources are modulated. This strategic difference is what we have conceptualized as the dichotomy between “mode-sensitive” and “mode-consolidated” neural profiles.

### 4.2 The evolution of neural strategy: from “mode-sensitive” to “mode-consolidated”

The adult brain possesses a remarkable capacity for reorganization following a stroke, compensating for functional loss primarily by enhancing activity within existing neural networks. A hallmark of this recovery process is the phenomenon where motor task execution elicits not only a widespread, bilateral activation of motor pathways but also the compensatory recruitment of sensory and secondary motor areas not typically engaged in the task (Calautti and Baron, 2003; Hannanu et al., 2017). Our findings validate this principle, showing that active-participation tasks engage a diffuse network far beyond the contralateral M1. We propose that the evolution from a “mode-sensitive” to a “mode-consolidated” state represents the macroscopic expression of this network reorganization over time. This process of “focusing” or consolidation, however, is not monolithic; it can follow two distinct trajectories. The optimal path involves a “normalization” of the activation pattern, gradually refocusing onto the lesioned hemisphere's original motor networks, a process that consistently predicts a better functional prognosis (Cheng et al., 2015; Ward, 2017). Alternatively, the brain may establish a “compensatory” recovery pathway, persistently relying on alternative neural resources, such as the contralesional hemisphere (Ward, 2005). While this form of compensation may be necessary for initial functional gains, it typically signifies a lower ceiling for ultimate recovery (Lotze et al., 2006).

This entire strategic evolution from a diffuse, effortful state to a more focal, efficient one aligns remarkably well with the classic theory of motor learning proposed by Fitts and Posner (1967) and Krakauer (2006), framing post-stroke recovery as a process of motor “re-learning” (Dayan and Cohen, 2011). The “mode-sensitive” state—observed in our subacute, low-function, and left-hemiplegic patients—mirrors the early cognitive and associative stages of learning. This aligns with the concept of “learning-driven” adaptive approaches, where the brain actively engages in strategy development and experimentation to overcome functional deficits (Egan et al., 2024). It is a period of intense, exploratory network remodeling characterized by high flexibility and a strong dependence on external cues to guide behavior. A more granular understanding of this state can be achieved through the lens of the adaptive network plasticity framework proposed by Hartwigsen and Volz (2021). They outlined three universal mechanisms the brain employs when facing disruption: (1) inter-hemispheric reorganization, recruiting homologous areas in the intact hemisphere for support; (2) inter-network interaction, dissolving functional boundaries to recruit adjacent specialized networks; and (3) enhancement of domain-general networks, increasing top-down cognitive control to compensate for lost automaticity (Hartwigsen and Volz, 2021). We posit that the “mode-sensitive” state is the direct neurophysiological manifestation of the brain actively and concurrently engaging all three of these plasticity mechanisms in an unsettled, exploratory phase (Cramer, 2008).

Conversely, the “mode-consolidated” state is analogous to the autonomous stage of motor learning, representing a new, stable equilibrium. This consolidation, however, is critically ambivalent. It can be the successful outcome of the “normalization” process, reflecting the re-establishment of efficient, focal neural pathways (Ward et al., 2003). Alternatively, it can be the consequence of maladaptive plasticity, where a less efficient compensatory strategy becomes entrenched and resistant to further change (Takeuchi and Izumi, 2012). This highlights the profound clinical importance of therapeutic intervention during the “mode-sensitive” phase, to guide the reorganization process toward a beneficial “normalization” rather than allowing it to settle into a suboptimal compensatory pattern. Our findings therefore characterize the core neural mechanism of post-stroke recovery as a dynamic evolution of activation strategy, a process that is not merely about changes in activation intensity but about a fundamental shift in the brain's approach to solving the problem of motor control.

### 4.3 Hemispheric specialization: the critical modulator of recovery strategy

One of the most profound findings of our study is the striking divergence in neural recovery strategies between patients with left and right hemiplegia. This clear dichotomy cannot be explained by a simple evolution from sensitive to consolidated states based on time or function alone; instead, it strongly suggests that hemispheric specialization is a decisive factor in determining the brain's entire approach to motor recovery. The inherent functional asymmetry of the cerebral hemispheres appears to fundamentally shape the available pathways for neuroplasticity, leading to distinct, side-dependent activation strategies. This observation aligns with research in healthy, right-handed individuals, which shows that executing a simple motor task with the non-dominant (left) hand elicits a significantly more bilateral and widespread pattern of brain activation compared to the more focal, contralateral activation seen during dominant (right) hand movement (Kim et al., 1993). This baseline asymmetry provides a crucial neurophysiological context for understanding the post-stroke brain.

We propose that the earlier and more pronounced “mode-consolidated” strategy observed in our patients with right hemiplegia (left hemisphere lesion) stems from the functional constraints imposed by injuring the dominant hemisphere. The left hemisphere is specialized for motor planning, sequencing, and analysis. When it is damaged, the brain's capacity for large-scale, inter-hemispheric functional transfer may be limited (Zemke et al., 2003). As research by Vidal et al. (2017) suggests, patients with left hemisphere lesions recruit significantly fewer neural resources from the contralesional (right) hemisphere during motor tasks. Consequently, their recovery may rely more heavily on the reorganization of peri-lesional areas within the damaged hemisphere (Vidal et al., 2017). This leads to a more constrained, focal, and less flexible compensatory strategy, which, while potentially effective, becomes consolidated more rapidly and appears as “mode-insensitive” in our analysis.

In stark contrast, the highly “mode-sensitive” profile of patients with left hemiplegia (right hemisphere lesion) reflects a fundamentally different and more dynamic recovery process, likely orchestrated by the intact, dominant left hemisphere. When the non-dominant right hemisphere is injured, the functionally powerful left hemisphere remains unscathed, potentially leading to two concurrent phenomena. First, according to the theory of interhemispheric inhibition imbalance, the intact left hemisphere may be “released” from transcallosal inhibition, leading to a state of heightened activity. This hyperactivity could, in turn, exert stronger inhibitory signals on the already vulnerable right hemisphere, potentially impeding its intrinsic capacity for reorganization (Dafotakis et al., 2008). Second, and perhaps more critically, the powerful left hemisphere may assume a higher-level, executive role in coordinating and planning the entire recovery process. This involves actively recruiting and modulating a widespread, bilateral network to explore and test various motor solutions, resulting in the more diffuse and dynamic activation patterns we observed (Grefkes and Fink, 2014). This constant strategic adjustment in response to external conditions—such as the different sensory cues from the robot—is the very essence of the “mode-sensitive” state (Vidal et al., 2017). It is this high degree of flexibility and responsiveness to the external environment that fundamentally defines their recovery strategy.

### 4.4 Rethinking lateralization: why mode sensitivity is a more robust biomarker

Intriguingly, our study did not observe consistent evidence for the classic “contralesional compensation” model, as we observed no significant hemispheric activation asymmetry in any of the stratified subgroups. This null finding does not necessarily refute the existence of contralesional compensation but rather highlights its complexity and inter-individual variability. The classic model may be an oversimplification (Dodd et al., 2017). Our heterogeneous sample likely includes patients employing diverse compensatory strategies—some relying on the contralesional hemisphere, others focusing on ipsilesional pathways (Buetefisch, 2015; Mansour et al., 2022). At the group level, these opposing strategies may neutralize each other, resulting in the observed bilateral balance. This underscores a critical methodological point: global lateralization indices can be misleading in heterogeneous cohorts. We therefore propose that “mode sensitivity,” which captures the dynamic responsiveness of the brain's entire strategic network, is a more robust and functionally relevant biomarker of an individual's neural recovery state than a simple static measure of hemispheric balance.

### 4.5 Clinical implications: a neurobiologically-informed framework for precision rehabilitation

**Figure 8:**
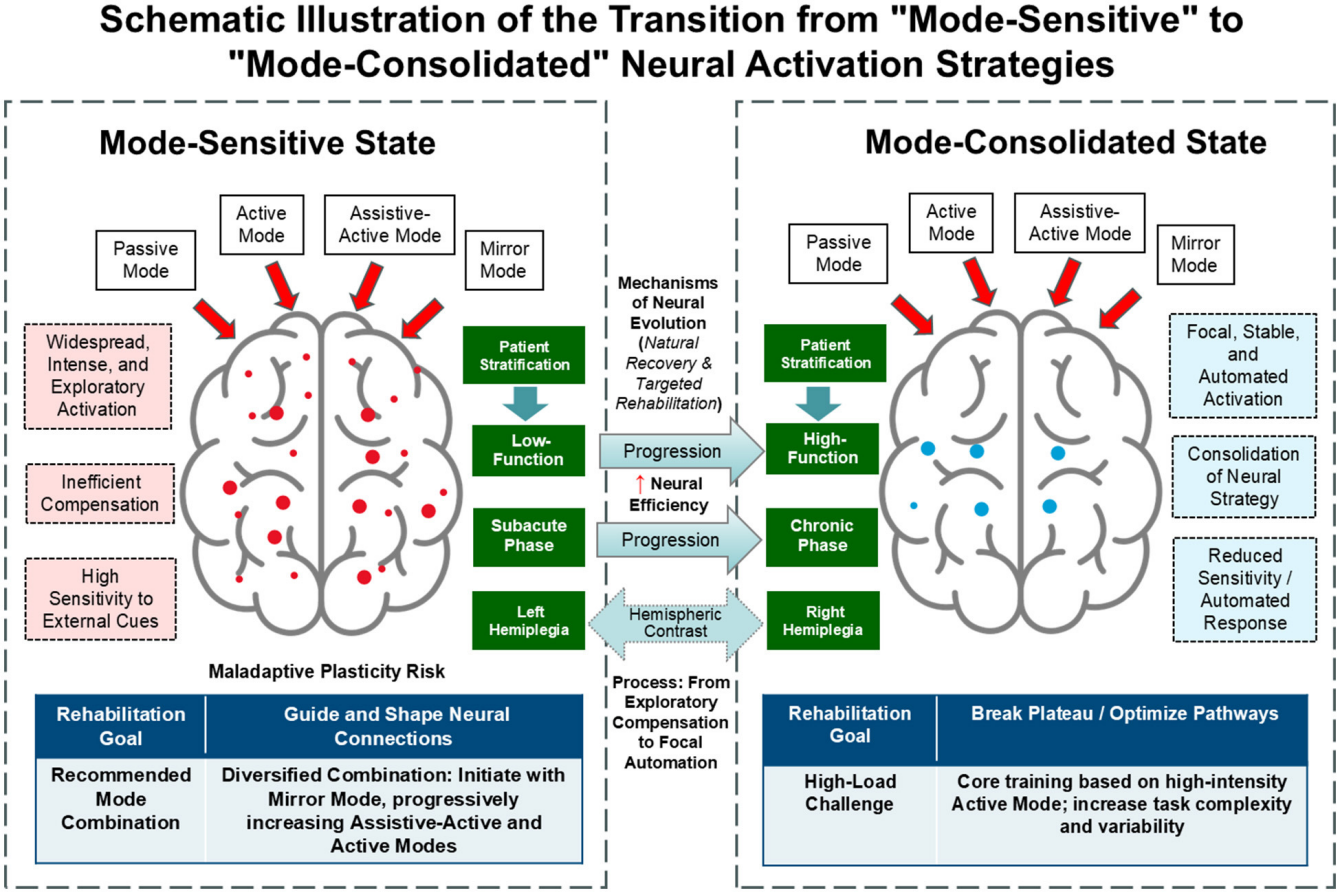
This conceptual model synthesizes the study's neuroimaging findings into a precision rehabilitation framework. Mode-Sensitive State (**Left Panel**): characteristic of patients with low function, in the subacute phase, or with left hemiplegia. This state is marked by widespread, intense, and exploratory cortical activation (red nodes), reflecting an inefficient compensatory network with high sensitivity to external cues. The recommended therapeutic goal is to “Guide and Shape” neural connections using a Diversified Combination of modes (initiating with Mirror mode and progressing to Assistive-Active). Mode-Consolidated State (**Right Panel**): characteristic of patients with high function, in the chronic phase, or with right hemiplegia. This state exhibits focal, stable, and automated activation patterns (concentrated blue nodes), reflecting the consolidation of neural strategy with reduced sensitivity to mode variations. The recommended therapeutic goal is to “Break the Plateau” by applying High-Load Challenges (high-intensity Active mode) to optimize established pathways. Center (Mechanisms of Neural Evolution): The central arrows illustrate the dynamic evolution of recovery. Solid arrows denote the Progression of neural efficiency correlated with time and functional improvement, while the dashed arrow highlights the distinct Hemispheric Contrast in recovery strategies between left and right hemiplegia.

The core findings of this study—the evolution of neural strategy from “mode-sensitive” to “mode-consolidated”—provide a clear, neuroscientifically-grounded roadmap for establishing truly individualized robot-assisted rehabilitation protocols. Our research strongly advocates for moving beyond a “one-size-fits-all” prescription and instead tailoring the selection and combination of training modes to the patient's functional status, chronicity, and, critically, the specific characteristics of their hemispheric lesion. This stratified approach, outlined in Figure 8, defines two distinct therapeutic philosophies guided by the patient's underlying neural strategy.

For patients in a “mode-sensitive” state—typically those in the subacute phase, with lower functional ability, or with left hemiplegia—the brain is in a state of heightened neuroplasticity and active exploration, but with limited voluntary motor capacity. The primary therapeutic goal for this group is to guide and shape the nascent neural connections. For patients with minimal or no voluntary movement, Mirror Mode serves as the ideal initial intervention. By leveraging the powerful visual input of the mirror neuron system, this mode can leverage the mirror neuron system to directly increase the excitability of the ipsilesional primary motor cortex (M1) even in the absence of an efferent motor command (Altschuler et al., 1999; Thieme et al., 2013; Saffin and Tohid, 2016). This provides a high-reward, low-threshold form of neural stimulation. Once the patient can generate even a flicker of motor intent, Assistive-Active Mode should be immediately introduced. This mode provides a critical function by precisely pairing the patient's weak efferent signal with the afferent sensory feedback of a successful, robot-assisted movement, creating a powerful Hebbian association that strengthens residual corticospinal pathways (Stefan et al., 2000; Krebs and Hogan, 2006). The initial phase of rehabilitation should therefore focus on a combination of “Mirror + Assistive” modes to facilitate, guide, and shape the formation of correct neural pathways.

Conversely, for patients who have transitioned to a “mode-consolidated” state—typically those in the chronic phase, with higher functional ability, or with right hemiplegia—the brain has already entered a more stable phase and is no longer sensitive to simple variations in training mode. The therapeutic goal for this group must be to “break the plateau” and further optimize the already-formed motor pathways. The most potent driver for experience-dependent neuroplasticity in this context is high-intensity, repetitive, and volitional movement (Nudo et al., 1996). Therefore, Active Mode should form the core of their rehabilitation. Since the brain is no longer sensitive to the mode itself, the therapeutic challenge must come from the task. This aligns perfectly with the “Challenge Point Framework,” which posits that learning is optimized only when the task provides a sufficient level of difficulty (Guadagnoli and Lee, 2004). Consequently, therapy should focus on a high-dose, high-intensity Active Mode regimen, with progressive increases in resistance, the introduction of cognitive dual-tasks, or the use of variable practice (e.g., changing movement targets, speeds, and trajectories) to continuously challenge the patient's motor control system (Newell, 2003; Seidler, 2004).

Furthermore, the strategy must be refined based on the side of hemiplegia. Patients with left hemiplegia, while highly “sensitive,” are also at greater risk of developing maladaptive plasticity due to the over-dominance of the intact left hemisphere (Murase et al., 2004; Riecker et al., 2010). Their diversified training program should be carefully designed to explicitly encourage and reward the participation of the affected (right) hemisphere. For patients with right hemiplegia, whose brains are more “consolidated,” the focus should be on reinforcement rather than change (Ward et al., 2003; Saur et al., 2006). Therapy should be implemented earlier and more intensively, focusing on high-repetition, function-oriented Active Mode training to solidify and optimize the more focal neural pathways that have already been formed.

### 4.6 Limitations and future directions

Despite its novel insights, this study has several limitations. The cross-sectional design captures only a single snapshot in time and precludes direct observation of the evolution from a sensitive to a consolidated state within individuals. Second, after stratification, the sample sizes within some subgroups were relatively small, which may limit statistical power. Third, the spatial resolution of fNIRS is inherently limited to cortical structures, preventing the analysis of key subcortical nodes in the motor network. Future research should prioritize longitudinal designs to validate the proposed evolutionary trajectory of neural strategies. Advanced functional connectivity analyses are also needed to elucidate the underlying network dynamics that distinguish these two states (Grefkes and Fink, 2014; Silasi and Murphy, 2014). Finally, our framework opens exciting possibilities for interventional studies, exploring whether non-invasive brain stimulation could be used to modulate a patient's neural state, potentially re-inducing a state of “mode sensitivity” in chronic patients to create a new window for therapeutic gains.

## 5 Conclusion

This study reveals that the core neural regulatory mechanism of post-stroke rehabilitation is characterized by a strategic evolution from a “mode-sensitive” to a “mode-consolidated” state of cortical activation. We have demonstrated that a patient's functional level, disease chronicity, and, most critically, the side of the hemispheric lesion are key modulators of this strategic progression. This discovery drives a paradigm shift in neurorehabilitation, moving the focus from simply “how much” the brain is activated to the more crucial question of “how” it activates. The future of precision rehabilitation should be guided by this principle, strategically tailoring therapeutic interventions to a patient's distinct neural profile: applying diversified, cue-rich stimuli to “mode-sensitive” individuals to guide neural reorganization, while applying high-load challenges to “mode-consolidated” individuals to break through functional plateaus and optimize established pathways.

## Data Availability

The datasets presented in this study can be found in online repositories. The names of the repository/repositories and accession number(s) can be found in the article/Supplementary material.
