# Supplementary material for: Modulation of cerebral activation strategies by training mode in stratified stroke cohorts: an fNIRS study
Source: Front Hum Neurosci. 2026 Mar 18;20:1767418. doi: 10.3389/fnhum.2026.1767418 (PMC13038619; doi:10.3389/fnhum.2026.1767418)
Supplement: Supplementary file 1 [file Table_1.docx]

Supplementary Material

Supplementary Table 1. MNI standard coordinates for 48 channels.

| Label CH | MNI Coordinates（x,y,z） | | | Brodmann Area | Percentage |
| --- | --- | --- | --- | --- | --- |
| CH1 | 61.12 | -51.28 | 46.28 | 40 - Supramarginal gyrus part of Wernicke's area | 0.8205 |
| CH2 | 48.3 | -49.42 | 58.77 | 40 - Supramarginal gyrus part of Wernicke's area | 0.8566 |
| CH3 | 52.79 | 44.55 | -9.73 | 46 - Dorsolateral prefrontal cortex | 0.4702 |
| CH4 | 38.26 | 64.29 | -4.54 | 10 - Frontopolar area | 0.4572 |
| CH5 | 25.81 | 68.16 | 13.42 | 10 - Frontopolar area | 0.9863 |
| CH6 | -37.18 | 64.78 | -2.42 | 10 - Frontopolar area | 0.607 |
| CH7 | -23.46 | 68.03 | 14.85 | 10 - Frontopolar area | 0.9965 |
| CH8 | -54.36 | 42.27 | -7.58 | 46 - Dorsolateral prefrontal cortex | 0.4067 |
| CH9 ( | -69.03 | -30.56 | 36.2 | 2 - Primary Somatosensory Cortex | 0.5662 |
| CH10 | -64.04 | -4.09 | 36.71 | 43 - Subcentral area | 0.465 |
| CH11 | 23.64 | -50.85 | 75.59 | 7 - Somatosensory Association Cortex | 0.4967 |
| CH12 | 36.61 | -51.31 | 69.91 | 7 - Somatosensory Association Cortex | 0.6926 |
| CH13 | 47.14 | 53.56 | 6.34 | 46 - Dorsolateral prefrontal cortex | 0.8502 |
| CH14 | 37.07 | 56.99 | 23.43 | 46 - Dorsolateral prefrontal cortex | 0.8729 |
| CH15 | 13.54 | 65.83 | 27.34 | 10 - Frontopolar area | 0.9301 |
| CH16 | -11.87 | 65.72 | 29.17 | 10 - Frontopolar area | 0.8381 |
| CH17 | -48.15 | 51.91 | 10.56 | 46 - Dorsolateral prefrontal cortex | 0.7849 |
| CH18 | -33.93 | 55.41 | 27.33 | 46 - Dorsolateral prefrontal cortex | 1 |
| CH19 | 32.74 | 0.54 | 66.76 | 6 - Pre-Motor and Supplementary Motor Cortex | 0.9399 |
| CH20 | 23.97 | -24.36 | 76.58 | 4 - Primary Motor Cortex | 0.6234 |
| CH21 | 36.68 | -23.49 | 72.1 | 4 - Primary Motor Cortex | 0.7023 |
| CH22 ( | 20.43 | 0.65 | 75.13 | 6 - Pre-Motor and Supplementary Motor Cortex | 1 |
| CH23 | 47.85 | 25.76 | 43.58 | 44 - pars opercularis_ part of Broca's area | 0.4082 |
| CH24 | 40.19 | 24.7 | 54.03 | 9 - Dorsolateral prefrontal cortex | 0.7908 |
| CH25 | -40.93 | 19.12 | 56.72 | 9 - Dorsolateral prefrontal cortex | 0.7119 |
| CH26 | -49.29 | 20.51 | 46.65 | 9 - Dorsolateral prefrontal cortex | 0.5959 |
| CH27 | -17.16 | 19.49 | 67.58 | 8 - Includes Frontal eye fields | 0.5887 |
| CH28 | -29.34 | 19.1 | 62.18 | 8 - Includes Frontal eye fields | 0.8679 |
| CH29 | -22.7 | -26 | 76.63 | 4 - Primary Motor Cortex | 0.7524 |
| CH30 | -20.94 | -2.99 | 76.48 | 6 - Pre-Motor and Supplementary Motor Cortex | 1 |
| CH31 | -36.99 | -27.79 | 71.33 | 4 - Primary Motor Cortex | 0.6986 |
| CH32 | -32.91 | -4.18 | 67.16 | 6 - Pre-Motor and Supplementary Motor Cortex | 1 |
| CH33 | 60.24 | -22.78 | 51.83 | 1 - Primary Somatosensory Cortex | 0.4887 |
| CH34 | 55.52 | 3.13 | 49.32 | 6 - Pre-Motor and Supplementary Motor Cortex | 0.9478 |
| CH35 | 44.02 | 2.69 | 60.07 | 6 - Pre-Motor and Supplementary Motor Cortex | 0.918 |
| CH36 | 49.16 | -21.88 | 65 | 3 - Primary Somatosensory Cortex | 0.4598 |
| CH37 | 69.59 | -24.64 | 35.3 | 2 - Primary Somatosensory Cortex | 0.6782 |
| CH38 | 64.17 | 1.58 | 33.55 | 6 - Pre-Motor and Supplementary Motor Cortex | 0.4844 |
| CH39 | 27.55 | 23.75 | 60.58 | 8 - Includes Frontal eye fields | 0.9887 |
| CH40 | 16.76 | 21.89 | 66.78 | 8 - Includes Frontal eye fields | 0.6829 |
| CH41 | -22.87 | -52.56 | 75.47 | 7 - Somatosensory Association Cortex | 0.6138 |
| CH42 | -36.27 | -54.44 | 68.27 | 7 - Somatosensory Association Cortex | 0.716 |
| CH43 | -47.13 | -54.27 | 58.46 | 40 - Supramarginal gyrus part of Wernicke's area | 0.8427 |
| CH44 | -59.06 | -56.6 | 46.37 | 40 - Supramarginal gyrus part of Wernicke's area | 0.5837 |
| CH45 | -49.22 | -29.37 | 65.53 | 3 - Primary Somatosensory Cortex | 0.456 |
| CH46 | -44.74 | -3.59 | 61.32 | 6 - Pre-Motor and Supplementary Motor Cortex | 0.844 |
| CH47 | -59.9 | -28.05 | 53.17 | 3 - Primary Somatosensory Cortex | 0.3113 |
| CH48 | -56.07 | -5.14 | 52.54 | 6 - Pre-Motor and Supplementary Motor Cortex | 0.6939 |

Note: The table lists the Montreal Neurological Institute (MNI) standard space coordinates (x, y, z) for the 48 fNIRS channels used in this study. The "Brodmann Area" column indicates the most probable Brodmann area and its corresponding anatomical name for each channel coordinate. The "Percentage" column represents the probability or confidence level that the channel coordinate falls within the specified Brodmann area.

Supplementary Table 2a. Comparison of Global Mean Activation during Active Mode between Left and Right Hemiplegia Groups

| Group | n | Mean ± SD | t-value | p-value |
| --- | --- | --- | --- | --- |
| Left Hemiplegia | 21 | 0.065 ± 0.047 | 0.787 | 0.436 |
| Right Hemiplegia | 20 | 0.052 ± 0.055 |  |  |

Note: ndependent samples t-test was used. Activation values are in units of ΔHbO (mmol/L*mm). SD = Standard Deviation.

Supplementary Table 2b. Intra-hemispheric and Inter-hemispheric Regional Asymmetry Analysis during Active Mode in the Left Hemiplegia Group (n=21)

| Comparison | Unaffected / Left | Affected / Right | Statistic | FDR p value |
| --- | --- | --- | --- | --- |
| Overall Hemispheric Activation | Mean = 0.060 | Mean = 0.069 | t = -1.467 | 0.158 |
| Corresponding Regional Activation | | | | |
| M1 | Mean = 0.055 | Mean = 0.080 | t = -1.525 | 0.381 |
| SMA | Mean = 0.063 | Mean = 0.057 | t = 0.678 | 0.893 |
| PMC | Mean = 0.069 | Mean = 0.075 | t = -0.596 | 0.893 |
| PSC | Mean = 0.063 | Mean = 0.088 | t = -1.723 | 0.381 |
| FP | Mean = 0.040 | Mean = 0.060 | t = -2.166 | 0.341 |
| DLPFC | Mean = 0.059 | Mean = 0.061 | t = -0.301 | 0.944 |
| FEF | Mean = 0.050 | Mean = 0.052 | t = -0.209 | 0.944 |
| SAC | Mean = 0.082 | Mean = 0.081 | t = 0.072 | 0.944 |

Note: Wilcoxon signed-rank test (reporting Medians) was used for all comparisons except for SAC, where a paired t-test (reporting Means) was used based on data distribution. All p-values are corrected for multiple comparisons using the Benjamini-Hochberg (FDR) procedure. Activation values are in units of ΔHbO (mmol/L*mm).

Supplementary Table 2c. Intra-hemispheric and Inter-hemispheric Regional Asymmetry Analysis during Active Mode in the Right Hemiplegia Group (n=20)

| Comparison | Unaffected / Left | Affected / Right | Statistic | FDR p value |
| --- | --- | --- | --- | --- |
| Overall Hemispheric Activation | Median = 0.054 | Median = 0.047 | Z = 77.000 | 0.312 |
| Corresponding Regional Activation | | | | |
| M1 | Median = 0.046 | Median = 0.049 | Z = 93.000 | 0.844 |
| SMA | Median = 0.030 | Median = 0.039 | Z = 84.000 | 0.731 |
| PMC | Mean = 0.051 | Mean = 0.048 | t = 0.199 | 0.844 |
| PSC | Median = 0.033 | Median = 0.039 | Z = 84.000 | 0.731 |
| FP | Mean = 0.085 | Mean = 0.044 | t = 3.010 | 0.058 |
| DLPFC | Mean = 0.072 | Mean = 0.058 | t = 1.160 | 0.731 |
| FEF | Mean = 0.035 | Mean = 0.028 | t = 0.759 | 0.731 |
| SAC | Mean = 0.065 | Mean = 0.061 | t = 0.279 | 0.844 |

Note: Wilcoxon signed-rank test (reporting Medians) or paired t-test (reporting Means) was used based on data distribution. All p-values are corrected for multiple comparisons using the Benjamini-Hochberg (FDR) procedure. Activation values are in units of ΔHbO (mmol/L*mm).

Supplementary Table 3a. Comparison of Global Mean Activation during Active Mode between High- and Low-Function Groups

| Group | n | Mean ± SD | t-value | p-value |
| --- | --- | --- | --- | --- |
| High-Function | 12 | 0.082 ± 0.061 | 1.452 | 0.165 |
| Low-Function | 29 | 0.054 ± 0.048 |  |  |

Note: ndependent samples t-test was used. Activation values are in units of ΔHbO (mmol/L*mm). SD = Standard Deviation.

Supplementary Table 3b. Intra-hemispheric and Inter-hemispheric Regional Asymmetry Analysis during Active Mode in the High-Function Group (n=12)

| Comparison | Ipsilesional | Contralesional | Statistic | FDR p value |
| --- | --- | --- | --- | --- |
| Overall Hemispheric Activation | Mean = 0.076 | Mean = 0.089 | t = -1.431 | 0.180 |
|  |  |  |  |  |
| Corresponding Regional Activation |  |  |  |  |
| M1 | Mean = 0.067 | Mean = 0.110 | t = -2.030 | 0.279 |
| SMA | Mean = 0.068 | Mean = 0.074 | t = -0.353 | 0.835 |
| PMC | Mean = 0.065 | Mean = 0.093 | t = -2.008 | 0.279 |
| PSC | Mean = 0.082 | Mean = 0.105 | t = -1.349 | 0.545 |
| FP | Mean = 0.097 | Mean = 0.078 | t = 0.945 | 0.730 |
| DLPFC | Mean = 0.070 | Mean = 0.079 | t = -0.631 | 0.835 |
| FEF | Mean = 0.050 | Mean = 0.057 | t = -0.378 | 0.835 |
| SAC | Median = 0.098 | Median = 0.084 | Z = 37.000 | 0.910 |

Note: Wilcoxon signed-rank test (reporting Medians) or paired t-test (reporting Means) was used based on data distribution. All p-values are corrected for multiple comparisons using the Benjamini-Hochberg (FDR) procedure. Activation values are in units of ΔHbO (mmol/L*mm).

Supplementary Table 3c. Intra-hemispheric and Inter-hemispheric Regional Asymmetry Analysis during Active Mode in the Low-Function Group (n=29)

| Comparison | Ipsilesional | Contralesional | Statistic | FDR p value |
| --- | --- | --- | --- | --- |
| Overall Hemispheric Activation | Mean = 0.053 | Mean = 0.054 | t = -0.116 | 0.909 |
|  |  |  |  |  |
| Corresponding Regional Activation |  |  |  |  |
| M1 | Median = 0.050 | Median = 0.054 | Z = 162.000 | 0.940 |
| SMA | Median = 0.032 | Median = 0.047 | Z = 199.000 | 0.940 |
| PMC | Mean = 0.059 | Mean = 0.061 | t = -0.227 | 0.940 |
| PSC | Mean = 0.045 | Mean = 0.057 | t = -0.882 | 0.940 |
| FP | Mean = 0.052 | Mean = 0.047 | t = 0.478 | 0.940 |
| DLPFC | Median = 0.065 | Median = 0.059 | Z = 209.000 | 0.940 |
| FEF | Mean = 0.041 | Mean = 0.041 | t = 0.075 | 0.940 |
| SAC | Mean = 0.063 | Mean = 0.062 | t = 0.098 | 0.940 |

Note: Wilcoxon signed-rank test (reporting Medians) or paired t-test (reporting Means) was used based on data distribution. All p-values are corrected for multiple comparisons using the Benjamini-Hochberg (FDR) procedure. Activation values are in units of ΔHbO (mmol/L*mm).

Supplementary Table 4a. Comparison of Global Mean Activation during Active Mode between Subacute and Chronic Phase Groups

| Group | n | Mean ± SD | t-value | p-value |
| --- | --- | --- | --- | --- |
| Subacute | 28 | 0.055 ± 0.051 | -1.221 | 0.235 |
| Chronic | 13 | 0.077 ± 0.056 |  |  |

Note: Independent samples t-test was used. Activation values are in units of ΔHbO (mmol/L*mm). SD = Standard Deviation.

Supplementary Table 4b. Intra-hemispheric and Inter-hemispheric Regional Asymmetry Analysis during Active Mode in the Subacute Group (n=28)

| Comparison | Ipsilesional | Contralesional | Statistic | FDR p value |
| --- | --- | --- | --- | --- |
| Overall Hemispheric Activation | Mean = 0.051 | Mean = 0.059 | t = -1.366 | 0.183 |
| Corresponding Regional Activation |  |  |  |  |
| M1 |  |  |  |  |
| SMA | Median = 0.057 | Median = 0.052 | Z = 145.000 | 0.516 |
| PMC | Median = 0.036 | Median = 0.042 | Z = 171.000 | 0.695 |
| PSC | Mean = 0.050 | Mean = 0.066 | t = -1.612 | 0.474 |
| FP | Mean = 0.045 | Mean = 0.073 | t = -2.158 | 0.320 |
| DLPFC | Mean = 0.044 | Mean = 0.045 | t = -0.099 | 0.922 |
| FEF | Mean = 0.059 | Mean = 0.055 | t = 0.518 | 0.695 |
| SAC | Mean = 0.036 | Mean = 0.043 | t = -0.711 | 0.695 |

Note: Wilcoxon signed-rank test (reporting Medians) or paired t-test (reporting Means) was used based on data distribution. All p-values are corrected for multiple comparisons using the Benjamini-Hochberg (FDR) procedure. Activation values are in units of ΔHbO (mmol/L*mm).

Supplementary Table 4c. Intra-hemispheric and Inter-hemispheric Regional Asymmetry Analysis during Active Mode in the Chronic Group (n=13)

| Comparison | Ipsilesional | Contralesional | Statistic | FDR p value |
| --- | --- | --- | --- | --- |
| Overall Hemispheric Activation | Mean = 0.079 | Mean = 0.076 | t = 0.238 | 0.816 |
|  |  |  |  |  |
| Corresponding Regional Activation |  |  |  |  |
| M1 | Mean = 0.061 | Mean = 0.093 | t = -1.634 | 1.000 |
| SMA | Mean = 0.068 | Mean = 0.063 | t = 0.344 | 1.000 |
| PMC | Mean = 0.085 | Mean = 0.075 | t = 0.693 | 1.000 |
| PSC | Mean = 0.073 | Mean = 0.072 | t = 0.058 | 1.000 |
| FP | Mean = 0.107 | Mean = 0.088 | t = 1.003 | 1.000 |
| DLPFC | Median = 0.056 | Median = 0.076 | Z = 45.000 | 1.000 |
| FEF | Mean = 0.059 | Mean = 0.051 | t = 0.520 | 1.000 |
| SAC | Mean = 0.089 | Mean = 0.084 | t = 0.221 | 1.000 |

Note: Wilcoxon signed-rank test (reporting Medians) or paired t-test (reporting Means) was used based on data distribution. All p-values are corrected for multiple comparisons using the Benjamini-Hochberg (FDR) procedure. Activation values are in units of ΔHbO (mmol/L*mm).
